# Impact of Patient Beliefs on Blood Pressure Control in the General Population: Findings from the Population-Based STAAB Cohort Study

**DOI:** 10.1155/2019/9385397

**Published:** 2019-07-28

**Authors:** Theresa Tiffe, Caroline Morbach, Viktoria Rücker, Götz Gelbrich, Martin Wagner, Hermann Faller, Stefan Störk, Peter U. Heuschmann

**Affiliations:** ^1^Institute of Clinical Epidemiology and Biometry, University of Würzburg, Germany; ^2^Comprehensive Heart Failure Center, University of Würzburg, Germany; ^3^Department of Medicine I, University Hospital Würzburg, Germany; ^4^Clinical Trial Unit, University Hospital Würzburg, Germany

## Abstract

*Background. *Effective antihypertensive treatment depends on patient compliance regarding prescribed medications. We assessed the impact of beliefs related towards antihypertensive medication on blood pressure control in a population-based sample treated for hypertension.* Methods. *We used data from the Characteristics and Course of Heart Failure Stages A-B and Determinants of Progression (STAAB) study investigating 5000 inhabitants aged 30 to 79 years from the general population of Würzburg, Germany. The* Beliefs about Medicines Questionnaire German Version (BMQ-D)* was provided in a subsample without established cardiovascular diseases (CVD) treated for hypertension. We evaluated the association between inadequately controlled hypertension (systolic RR >140/90 mmHg; >140/85 mmHg in diabetics) and reported concerns about and necessity of antihypertensive medication.* Results. *Data from 293 participants (49.5% women, median age 64 years [quartiles 56.0; 69.0]) entered the analysis. Despite medication, half of the participants (49.8%) were above the recommended blood pressure target. Stratified for sex, inadequately controlled hypertension was less frequent in women reporting higher levels of concerns (OR 0.36; 95%CI 0.17-0.74), whereas no such association was apparent in men. We found no association for specific-necessity in any model.* Conclusion. *Beliefs regarding the necessity of prescribed medication did not affect hypertension control. An inverse association between concerns about medication and inappropriately controlled hypertension was found for women only. Our findings highlight that medication-related beliefs constitute a serious barrier of successful implementation of treatment guidelines and underline the role of educational interventions taking into account sex-related differences.

## 1. Introduction

Although high blood pressure is a well modifiable risk factor, hypertension control remains unsatisfactory in the general population in Germany [1].

The reasons for noncompliance to pharmacotherapeutic recommendations are multifaceted. It has been proposed that patients' disease-modified beliefs may have a greater impact on medication adherence than external barriers as, e.g., cost, access to refills, logistic issues, or hospitalization [2–6]. The Necessity-Concern-Framework demonstrated that, in patients suffering from a variety of long-term conditions including hypertension, adherence to therapy was influenced by implicit judgments regarding personal needs for (i.e., necessity beliefs) and concerns about the prescribed medication [7]. The present investigation into medication-related beliefs focused on adherence measurement scales as primary outcome, rather than on objectively measured clinical outcomes, such as hypertension control.

We aimed to evaluate the impact of individuals' beliefs of* specific-concerns *and* specific-necessity *towards prescribed antihypertensive medication on inadequate blood pressure control and potential sex-related differences in a population-based sample of the general population without established cardiovascular disease (CVD) treated for hypertension.

## 2. Methods

We present data of the Characteristics and Course of Heart Failure Stages A–B and Determinants of Progression (STAAB) cohort study. The detailed study protocol and data collection have been published previously [8].

The* Beliefs about Medicines Questionnaire* developed by Horne was validated in a broad range of diseases, including cardiac samples, e.g., hypertension, to assess patient beliefs towards medication [9–11]. We used the* Beliefs about Medicines Questionnaire specific* subscales of the German version (BMQ-D) to assess personal beliefs about* specific-concerns *(6 items; e.g., danger of dependence or long-term toxicity) and* specific-necessity* of their prescribed medication (5 items; e.g., importance of prescribed therapy regarding their health status now and in the future), ranging from 1 “strongly disagree” to 5 “strongly agree”. Higher scores indicate higher levels of concerns (range 0-30) or necessity (range 0-25) [12].* A priori*, a set of covariates was identified potentially related to blood pressure control including sociodemographic status (age, sex, and education), current smoking, self-reported diabetes, and body mass index (BMI) [13–16]. Sociodemographic status, self-reported diabetes, and information on current smoking were obtained via face-to-face interview. Blood pressure values were given as median of up to three valid measurements in sitting position. Study physicians assessed self-reported history of CVD (coronary artery disease, peripheral artery disease and stroke) and medication intake. BMI was calculated divided weight in kilograms by the square of height in meters. Target blood pressure of <140/90 mmHg (<140/85 mmHg in diabetics) and overweight (BMI >25 kg/m^2^) were applied according to recent* European Guidelines on CVD prevention in clinical practice* (version 2016) [17]. Antihypertensive pharmacotherapy included substances classified in ATC group C01 up to C10.

## 3. Data Analysis

We calculated median (quartiles) for continuous variables and proportions for categorical variables. In univariable analyses, Fisher's exact test or *χ*^2^- test for categorical and binary variables were used as appropriate. Due to right-skewed distribution of the BMQ-D* specific-concerns *scale, both specific subscales were dichotomized at the median score obtained per scale. As a sensitivity analysis, we also included the subscales as a continuous variable to the model. We performed multivariable logistic regression analysis to assess the association of* specific-concerns* and* specific-necessity* on inadequately controlled hypertension adjusted for sociodemographic status (age, sex, education), overweight (BMI >25 kg/m^2^), self-reported smoking status, and self-reported diabetes. Possible interactions between sex and beliefs regarding prescribed antihypertensive medication were assessed by adding terms of interaction to the regression model. In case of significant terms of interaction, univariable logistic regression (crude odds ratio [OR] with 95% confidence intervals [CI]) on inadequately controlled hypertension stratified for sex was performed and then adjusted for all covariables (adjusted OR). P-values <0.05 were considered statistically significant. Analyses were performed with IBM SPSS Statistics 23 (IBM® SPSS® Statistics Version 23).

## 4. Results

**Figure 1:**
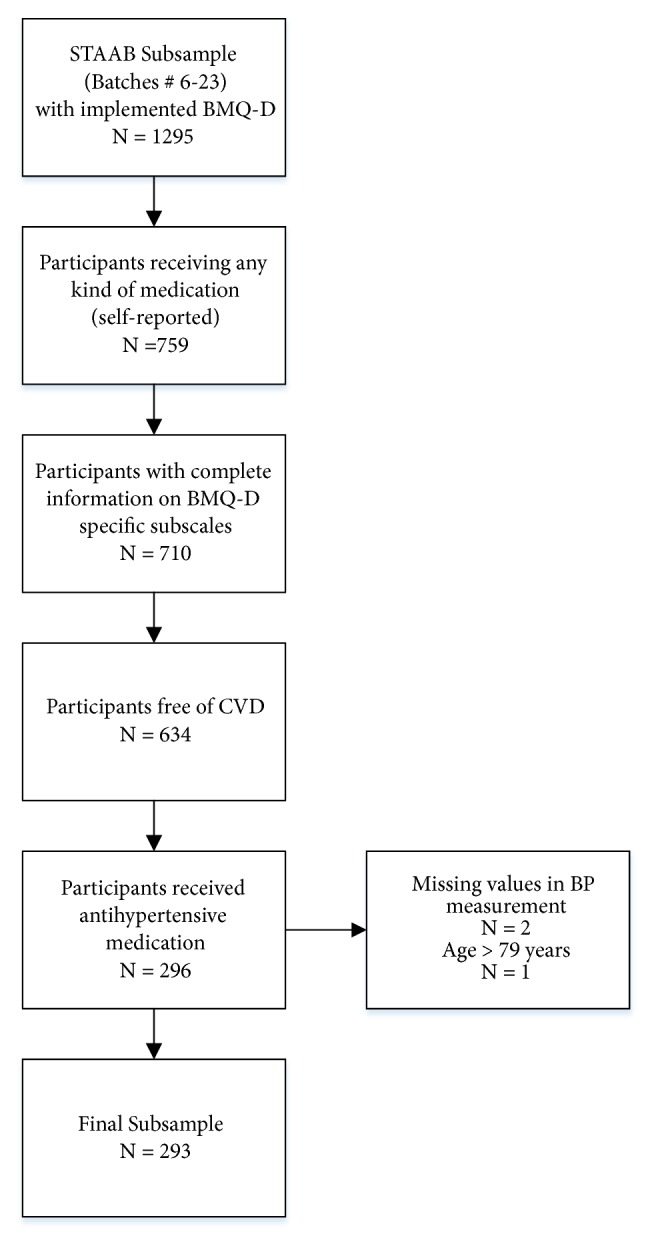
*Sample Selection*. BMQ-D, Beliefs about Medicines Questionnaire (German version); CVD, cardiovascular disease; BP, blood pressure.

We applied the BMQ-D in a sex- and age-stratified subsample of 1295 participants in STAAB recruited via protocol-defined send-out waves (batches) 6 to 23. Of 759 participants receiving any kind of medication, 710 (93.5%) had complete information on both specific subscales. Of those, 634 were free of self-reported CVD, and 296 out of those received antihypertensive medication; 3 participants with missing values in blood pressure measurement or age older than 79 years were excluded. Therefore, 293 participants free of CVD taking antihypertensive drugs and with complete information on BMQ-D specific subscales entered the analyses (Figure 1).

The median age of the sample was 64 years (quartiles 55.0; 69.0), 49.5% were women (Table 1), and the median blood pressure was 137.5/88.0 mmHg (women: 134.5/79.6 mmHg; men: 139.8/82.8 mmHg). Despite antihypertensive medication, half of the participants (49.8%) revealed a blood pressure value above the recommended target with a preponderance of men (57.5% vs. 42.5%, respectively).

Regarding the BMQ-D, the median score was 12.0 (9.0; 16.0) for* specific-concerns* and 17.0 (14.0; 20.5) for* specific-necessity*. More than 20% of women with normotensive blood pressure under medication reported concerns about dependency compared to 4.5% with blood pressure values above 140/90 mmHg. In the multivariable logistic regression analysis identifying factors associated with hypertension control, the interaction between specific-concerns and sex was statistically significant (p=0.02). Thus, further analyses were stratified for sex. In the adjusted multivariable regression model for women, inadequately controlled hypertension was less frequent in subjects expressing higher levels of concerns with regard to their prescribed antihypertensive medication (adjusted OR 0.36; 95%CI 0.17-0.74). Sensitivity analysis showed a similar effect for the continuous* specific-concern* subscale: adjusted OR per scale point 0.92; 95%CI 0.85-0.99. By contrast, in the univariable regression model for men, smoking was positively associated with better treatment control (crude OR 0.34; 95%CI 0.14-0.87). However, this effect disappeared after adjustment for other covariables: adjusted OR 0.43; 95%CI 0.16; 1.13. No statistically significant associations were found for* specific-necessity* in any model (Table 2).

## 5. Discussion

The current study observed insufficient blood pressure control in about half of the patients with a preponderance for men. This is in line with previous reports and [18] constitutes a major challenge for prevention of cardiovascular diseases.

The median BMQ scores for* specific-concerns* of 17.0 and* specific-necessity* of 12.0 were lower when compared to previous reports, which might be due to potentially healthier individuals with less comorbidity in our population-based sample [2, 3].

Potential sex-related differences in medication-related beliefs have been controversially discussed in previous studies [2, 19, 20]. Previous, qualitative studies showed that individuals, who do not perceive hypertension as a serious problem, have less controlled hypertension [21, 22]. In our study, we observed marked sex-related differences of such beliefs regarding inappropriate blood pressure control. Being less concerned about the prescribed antihypertensive medication was associated with worse blood pressure control in women, but not in men. In this context, considering that our finding was limited to women might indicate that adherence in female participants treated for hypertension is worse if their health perception is positive.

On the other hand, a study by Theunissen et al. showed that discussing cognitive or emotional illness representations (i.e., a process where patients are made aware of the significance of their health threat) or action plans regarding adherence to medical advice in a patient-provider-interaction led to more concerns about medication and increased patients' wish to follow lifestyle recommendations. This may additionally have strengthened their aversion to medication use [23]. Thus, illness representations may be both an indicator for better adherence and increased concerns. Therefore, well-informed patients knowing about the long-term consequences of uncontrolled hypertension might show better adherence to their medication regimes, despite higher concerns about medications.

In men, we did not find an association between specific scales of the BMQ-D and blood pressure targets. However, smoking was a factor associated with better treatment control in the unadjusted model, but the effect disappeared after adjustment for the other covariables, possibly due to the limited size of the sample. One study reported that more men than women believed in the serious longer-term consequences of hypertension [2]. Because of their higher CVD risk, male smoking participants with their additional cardiovascular risk factor of hypertension might be more aware of their blood pressure. Therefore, they might at least have their blood pressure treated appropriately if they cannot stop smoking.

No significant association was found for blood pressure control and the BMQ-D* specific-necessity* subscale. A systematic review by Horne and colleagues reported also no statistically significant association regarding necessity and adherence to antihypertensive medication [7]. There may be a link between the necessity of taking prescribed medication and the presence or absence of symptoms. When suffering from a predominantly asymptomatic condition such as hypertension, the benefits may be imperceptible to the participants [2, 9, 24]. Therefore, it is challenging for physicians to advise patients with hypertension to take their medication as prescribed because they tend to underestimate the long-term benefits of adherence to medication [2, 3, 12, 25]. Furthermore, in a condition that is mainly asymptomatic, medication-related side effects may more readily be perceived as unacceptable [2].

Recent studies have shown that educational interventions in hypertensive patients are able to influence the awareness of hypertension as a modifiable condition and the knowledge about prescribed therapy leading into a significant reduction in blood pressure values [26, 27]. The role of educational interventions on medication-related beliefs taking into account sex-related differences needs to be further investigated.

## 6. Limitations

There are limitations of our study. We report data from selected participants diagnosed with hypertension from an age- and sex-stratified population-based study with potentially limited generalizability to other populations and disease groups. Furthermore, our study may include healthier subjects with a higher proportion of asymptomatic blood pressure and less severe forms of hypertension due to the voluntary participation. Thus, medication-related beliefs might be underestimated. In addition, single-occasion measurements for hypertension may lead to false positive associations despite standardized measurements. Furthermore, we had no information about the number and dose of antihypertensive medication taken per day as well as the intensity of physicians' supervision, adherence to medication, and counselling that might influence medication-related beliefs. Last, the multivariable model only accounted for measured factors leaving room for residual confounding.

## 7. Conclusion

In contrast to previous studies examining medication-related beliefs towards adherence as a primary outcome, we assessed the association of medication-related beliefs with a clinical outcome, i.e., blood pressure control in a population-based sample. In our study, lower concerns about prescribed medication were associated with worse blood pressure control in women, but not in men. Our findings highlight the importance that medication-related beliefs constitute a serious barrier of successful implementation of treatment guidelines and underline the role of educational interventions taking into account sex-related differences. The unexpected finding of an inverse association regarding concerns about medication and blood pressure control needs to be replicated and further investigated in different populations with clinical outcomes.

## Figures and Tables

**Table 1:** Characteristics of the study population stratified according to achieved or not achieved recommended blood pressure target.

	N_Total_	Target achieved ^1^	Target not achieved	P-value
	*293*	*147 (50.2)*	*146 (49.8)*	
Gender				0.02
Female	145 (49.5)	83 (56.5)	62 (42.5)	
Male	148 (50.5)	64 (43.5)	84 (57.5)	
Age in years				0.49
30-39	4 (1.4)	3 (2.0)	1 (0.7)	
40-49	36 (12.3)	18 (12.2)	18 (12.3)	
50-59	52 (17.7)	25 (17.0)	27 (18.5)	
60-69	131 (44.7)	71 (48.3)	60 (41.1)	
70-79	70 (23.9)	30 (20.4)	40 (27.4)	
Education				0.26
primary	107 (36.5)	59 (40.1)	48 (32.9)	
secondary	94 (32.1)	48 (32.7)	46 (31.5)	
tertiary	92 (31.4)	40 (27.2)	52 (35.6)	
Current smoking	35 (11.9)	22 (15.0)	13 (8.9)	0.11
Self-reported diabetes	50 (17.1)	25 (50.0)	25 (50.0)	0.98
BMI > 25 kg/m^2^	237 (82.3)	122 (84.1)	115 (80.4)	0.41
BMQ, n (%), ≥ median				
Specific - Concerns	164 (56.0)	90 (61.2)	74 (50.7)	0.07
Specific - Necessity	151 (51.5)	78 (53.1)	73 (50.0)	0.60

Legend: data are count (percent). Analyses are restricted to patients without missing values in respective variables.

BMI, body mass index; BMQ, Beliefs about Medicine Questionnaire.

^1^ Recommended target for treated hypertension: ≤ 140/90 mmHg and for diabetics: ≤ 140/85 mmHg.

**Table 2:** Crude and adjusted ORs (95%-CI) for failure to reach blood pressure target in women and men.

Variables	Women				Men			
	Crude OR (95%CI)	P-value	Adjusted OR (95%CI)	P-value	Crude OR (95%-CI)	P-value	Adjusted OR (95%-CI)	P-value
*Age in years, n (%)*		0.72		0.63		0.26		0.35
≤ 49	1		1		1		1	
50-59	1.39 (0.40; 4.80)		1.34 (0.34; 5.30)		1.08 (0.35; 3.32)		1.53 (0.45; 5.21)	
60-69	0.82 (0.27; 2.46)		0.74 (0.23; 2.43)		1.13 (0.44; 2.90)		1.39 (0.51; 3.76)	
70-79	0.96 (0.29; 3.18)		1.15 (0.31; 4.27)		2.50 (0.84; 7.40)		2.75 (0.87; 8.67)	

*Education*		0.53		0.65		0.43		0.24
Tertiary	1		1		1		1	
Secondary	1.02 (0.44; 2.35)		1.06 (0.43; 2.64)		0.64 (0.27; 1.47)		0.45 (0.17; 1.18)	
Primary	0.68 (0.29; 1.60)		0.72 (0.28; 1.90)		0.64 (2.30; 1.37)		0.57 (0.24; 1.36)	
*Current smoking*		0.94		0.62		0.02		0.09
No	1		1		1		1	
Yes	0.96 (0.29; 3.16)		0.71 (0.18; 2.77)		0.34 (0.14; 0.87)		0.43 (0.16; 1.13)	
*Self-reported diabetes*		0.22		0.27				0.27
No	1		1		1		1	
Yes	1.74 (0.72; 4.19)		1.72 (0.66; 4.51)		0.60 (0.25; 1.40)		0.56 (0.22; 1.43)	
*Body Mass Index*		0.38		0.45		0.57		0.88
BMI ≤ 25 kg/m^2^	1		1		1		1	
BMI > 25 kg/m^2^	0.70 (0.31; 1.57)		0.72 (0.30; 1.71)		0.76 (0.29; 1.96)		1.09 (0.38; 3.11)	
*BMQ-Concerns*		< 0.01		< 0.01		0.77		0.57
Concerns ≤ median	1		1		1		1	
Concerns > median	0.37 (0.19; 0.73)		0.36 (0.17; 0.74)		1.11 (0.57; 2.13)		1.23 (0.60; 2.56)	
*BMQ-Necessity*		0.24		0.32		0.42		0.37
Necessity ≤ median	1		1		1		1	
Necessity > median	0.67 (0.34; 1.30)		0.68 (0.32; 1.45)		1.31 (0.68; 2.52)		1.38 (0.68; 2.82)	

Data are odds ratios (OR) with 95% confidence intervals (CI). For adjustment procedure refers to Method.

## Data Availability

All data necessary for interpreting the study results and supporting its conclusions are included in the present publication. The authors have full control of all primary data. Individual patient data are available upon request from STAAB principal investigators in accordance with local data security restrictions and ethics recommendations.
